# Receptor Tyrosine Kinases in Development: Insights from *Drosophila*

**DOI:** 10.3390/ijms21010188

**Published:** 2019-12-26

**Authors:** Sarah Mele, Travis K. Johnson

**Affiliations:** School of Biological Sciences, Monash University, Clayton, VIC 3800, Australia

**Keywords:** receptor tyrosine kinase (RTK), growth factor, cytokine, *Drosophila melanogaster*, cell signaling, cell fate

## Abstract

Cell-to-cell communication mediates a plethora of cellular decisions and behaviors that are crucial for the correct and robust development of multicellular organisms. Many of these signals are encoded in secreted hormones or growth factors that bind to and activate cell surface receptors, to transmit the cue intracellularly. One of the major superfamilies of cell surface receptors are the receptor tyrosine kinases (RTKs). For nearly half a century RTKs have been the focus of intensive study due to their ability to alter fundamental aspects of cell biology, such as cell proliferation, growth, and shape, and because of their central importance in diseases such as cancer. Studies in model organisms such a *Drosophila melanogaster* have proved invaluable for identifying new conserved RTK pathway components, delineating their contributions, and for the discovery of conserved mechanisms that control RTK-signaling events. Here we provide a brief overview of the RTK superfamily and the general mechanisms used in their regulation. We further highlight the functions of several RTKs that govern distinct cell-fate decisions in *Drosophila* and explore how their activities are developmentally controlled.

## 1. The Receptor Tyrosine Kinase Protein Superfamily

Receptor tyrosine kinases (RTKs) play essential roles in the cellular communication network that orchestrates the development of metazoans. They are a major class of enzyme-coupled cell surface receptors activated when bound by extracellular signals from the environment such as growth factors, cytokines, and hormones. Initially, RTKs were discovered in the 1970s as the key factors responsible for transducing several potent growth and proliferative signals, including nerve growth factor (NGF), epidermal growth factor (EGF), and insulin (for review see [1]). Since then, bioinformatic analysis has identified thousands of RTKs across eukaryotes. Each can be classified into one of 20 subfamilies based on structural elements and their homology with the founding mammalian representatives [2].

**Figure 1:**
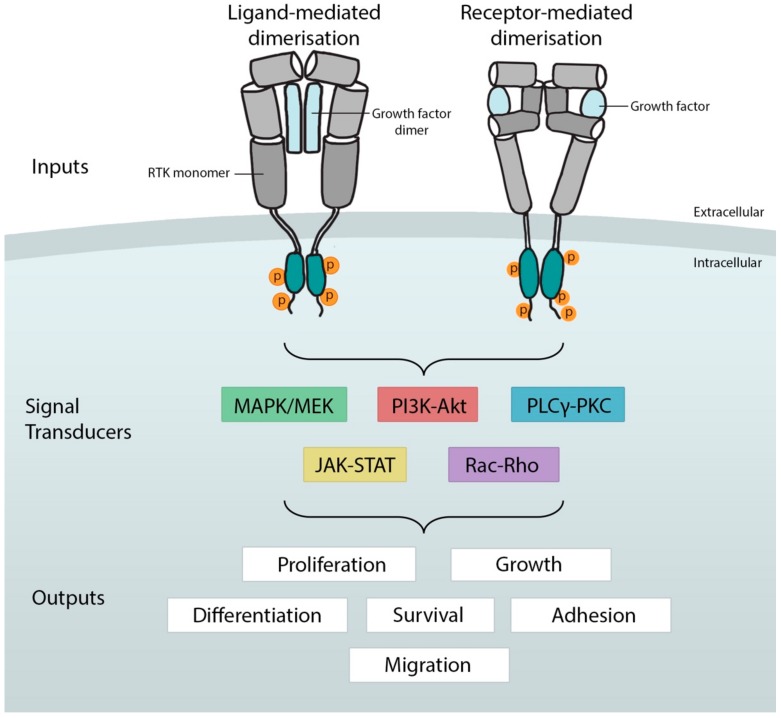
General overview of receptor tyrosine kinase activation, signaling, and the cell-fate decisions they influence. The binding of growth factors (inputs) in the extracellular milieu induces conformation changes in the receptor monomer that enables dimerization. Enzymatic autophosphorylation (circled p) by intracellular tyrosine kinase domains in *trans* results in recruitment of one or more signal transduction cascades. These relay the signal to effectors that determine cell fates (outputs). Mitogen-activated protein kinase, MAPK; phosphatidylinositol 3-kinase–protein kinase B, PI3K–Akt; phospholipase C gamma–protein kinase C, PLCgamma–PKC; Janus kinase and signal transducer and activator of transcription, JAK–STAT.

While members of the RTK protein superfamily are best known for roles in driving cell proliferation, they also play critical roles in eukaryotic development and homeostasis. These include the patterning of cells and tissues [3], the control of cell shape changes for migration and morphogenesis [4], cell and organ/tissue growth control [5,6], and the maintenance and survival of both developing and adult tissues [7,8] (Figure 1).

With their central importance in cellular events it is not surprising that RTK dysregulation is a major cause of disease. The aberrant activation of various RTKs is observed in nearly all forms of human cancer [9], and as such, these proteins are the targets of significant efforts to produce effective pharmacological inhibitors [10,11]. Beyond cancer, RTK signaling has been causally linked to diabetes [12], inflammation [13], angiogenesis [14], and numerous developmental syndromes (for review, see [15]). The roles of RTKs in human disease have been covered extensively elsewhere and will not be discussed here (see e.g., [16]).

### 1.1. RTK Structure, Function, and Signaling

RTKs are transmembrane glycoproteins that reside at the cell surface, where they catch growth factors from the extracellular milieu and subsequently transmit a signal to the inside of the cell via enzymatic phosphorylation [2]. The general structure of an RTK is defined by a variable extracellular ligand binding (ecto)domain, a hydrophobic single-pass transmembrane helix, and an intracellular protein tyrosine kinase domain (TKD). Ectodomains comprise a modular series of domains that permit interactions with distinct ligands (multiple ligands in many cases), regulatory cofactors, and other receptors [17]. In contrast, the intracellular portion of RTKs varies little and most commonly only comprises a single highly conserved TKD. Variations on this exist, however, including a split TKD (into two parts), catalytically inactive TKDs (e.g., RYK family and ErbB3 [18]), and by the presence of additional intracellular ancillary domains (e.g., the sterile alpha motif in human Eph receptors [19]). The insulin receptor subfamily is the most notable exception deviating from the prototypical RTK structure. Members of this family form as a heterotetramer composed of two disulphide linked heterodimers, rather than a single chain as is observed for members of other RTK subfamilies [20]. Due to the conserved nature of the TKD, it has been utilized extensively for identification of new RTKs, as well as their classification within the superfamily [21,22]. 

Ligand-induced dimerization is widely held as the canonical mechanism by which RTKs are activated. Dimerization occurs when a ligand and its RTK monomer associate and a conformational change is induced that permits the recruitment of a second receptor monomer to the complex (for review see [23]. More recently, an alternative model of has emerged whereby the RTK dimer (such as TrkA) exists in the absence of ligand [24]. Here, it is thought that ligand-binding is sufficient to invoke the conformational change necessary for RTK activation. In terms of ligand-binding, RTKs like TrkA, for example, use a ‘ligand-mediated’ mode, whereby a bivalent ligand (e.g., an NGF dimer) binds the two receptors simultaneously [25]. In other RTKs, such as EGFR (ErbB family), activation is ‘receptor-mediated’, meaning that ligand binding drives receptor–receptor interactions without ligand–ligand interactions [26]. There are also RTKs like the Fibroblast growth factor receptor (FGFR) that require cofactors in addition to ligand binding (e.g., heparin-like molecules [27,28]). 

Ligand-binding triggers the juxtaposition of the cytoplasmic TKDs, which in turn results in autophosphorylation in *trans* of tyrosine residues in the TKD activation loops. This serves to stabilize the kinase in an open and active conformation. Additional autophosphorylation of tyrosine residues in the juxtamembrane and carboxy-terminal regions control the recruitment of effector proteins that carry SRC-Homology 2 (SH2) or phosphotyrosine-binding (PDB) domains [29]. For example, Grb2 binds to phosphorylated tyrosines on active RTKs, allowing its translocation to the plasma membrane where it activates the membrane-bound G-protein Ras. Thus, these proteins serve to connect RTK phosphorylation to downstream signaling cascades [30].

The most common cascades employed to transmit the signal from RTKs are the mitogen-activated protein kinase (MAPK) cascades, PLCgamma, phosphatidylinositol 3-kinase (PI3K), and Janus Kinase and Signal Transducer and Activator of Transcription (JAK/STAT) signaling pathways (Figure 1). The MAPK cascades include the extracellular signal-related kinases (ERK1/2), c-Jun N-terminal kinases (JNKs) and P38-MAPKs [31]. Generally, ERKs are moderators of cell proliferation, growth and differentiation through transcriptional targets, while JNKs and p38 MAPKs respond to inflammatory cytokines and environmental stress [32]. 

In addition to the MAPK pathway, several RTKs including the Insulin receptor family use the PI3K-Akt-TOR signaling axis to modulate general protein synthesis, cell cycle progression, and inhibit apoptosis via S6K and/or the forkhead transcription factor FOXO [33,34]. Other transduction routes have also been described, for example, VEGFR2 can activate the PLCgamma pathway leading to phosphorylation of RAF and signaling via MAPK-ERK [35]. The JAK/STAT pathway also plays prominent roles in cell proliferation [36]. This pathway has been observed to bolster RTK signal outputs and mediate crosstalk between other pathways. Some examples of this include *Drosophila* EGFR [37,38], PDGFR [39], and *Drosophila* Torso [40]. 

### 1.2. General Mechanisms That Control RTK Activity in Development

The events described above are programmed to take place at precisely controlled locations and times both during development and in adult tissue homeostasis. Indeed, the spatiotemporal regulation of receptor abundance, as well as the availability and density at the membrane are all major determinants of RTK output [41]. Receptor location is not only important at the level of the cell and tissue types that express the RTK on their surface, but also to specific plasma membrane compartments. For example, in polarized cells basolateral and apical sides are distinct and separated by physical barriers (e.g., adherens and tight junctions), and thus populations of RTKs can be directed to either and their activity restricted. At the nanometer-scale, RTKs like other membrane integral proteins, occupy distinct plasma membrane microdomains such as caveolae and lipid-rafts [42,43]. These domains influence the spatial organization of RTKs in the membrane and thus the propensity with which active dimers or clusters can form.

Ligand location and abundance must also be coordinated with that of the receptor to ensure that the desired output is both achieved and robust to perturbation [44]. Over four decades of extensive study has revealed that the spatiotemporal control of ligands occurs in almost every conceivable way. Some of the more prominent mechanisms include localized and transient tissue expression, specialized intracellular trafficking routes, regulated secretion, and numerous post-translational modifications such as proteolytic processing, oligomerization, and glycosylation [45,46]. Additional points of spatiotemporal regulation occur once the ligand(s) has complexed with and activated its cognate receptor. For example, endocytic internalization of the signaling receptor oligomer can lead to rapid signal attenuation via lysosomal degradation, its maintenance, or its further enhancement by ligand–receptor decoupling and the recycling of receptors back to the membrane [47]. Further regulation via feedback loops are known to enhance or diminish signaling, too. The best characterized of these operate via the transcriptional upregulation of genes that encode modulators of upstream signaling components, including the RTK and ligands themselves [48,49,50].

A great deal of these mechanistic insights have been gained through studies of RTKs and their ligands in both cell culture systems and in model organism. In particular, genetic and functional analyses of RTKs in models, including the mouse, fruit fly, and nematode worm have provided the crucial contextual links for understanding how these proteins serve to control processes that underlie development. Studies of these organisms have revealed novel insights at all levels of RTK regulation and identified many of the components that underpin these mechanisms [51,52,53,54,55,56]. In the following sections, we will focus on the *Drosophila* RTKs and highlight the general mechanisms by which they are controlled.

## 2. *Drosophila melanogaster* as a Model to Study RTK Function in Development

Studies in the model organism *Drosophila melanogaster* have contributed substantially to our present understanding of RTK function in the context of development. This is largely owing to its genetic tractability, speed of life-cycle, and the strong conservation that exists between *Drosophila* and humans, which extends to most of the RTK families and the downstream intracellular signaling pathways [57,58]. A further advantage of using *Drosophila* for studying RTKs is the low level of within family complexity; the human genome encodes ~58 RTKs across 20 families, whereas the *Drosophila* genome encodes 20 known RTKs with single representatives for 11 of the 20 mammalian families (Table 1). This is particularly desirable since mammalian RTKs are known to form heterodimers with other family members (e.g., HER2 and ErbB [59]) to yield distinct and complex ligand-binding and signaling characteristics.

Akin to their mammalian counterparts, *Drosophila* RTKs play critical roles in all aspects of development, including differentiation and tissue patterning, morphogenesis, cell growth, and proliferation. There are too many developmental events that involve RTKs to cover all in sufficient detail here, so we have chosen to highlight several that have and continue to be highly informative for our broader understanding of RTK control in the context of animal development. For reference, a summary of *Drosophila* RTKs, their roles at each life-stage, and their cognate ligands involved are provided in Table 2. 

## 3. Epidermal Growth Factor Receptor

The epidermal growth factor receptor (EGFR) is the only *Drosophila* member of the EGFR/ErbB family of RTKs. It plays a multitude of roles during development, including patterning across both the dorsoventral axis and neuroectoderm during early embryogenesis, controlling the survival of glia during neurogenesis, differentiation and proliferation in the imaginal discs and brain, and in the ovary, where EGFR signaling patterns follicle cells and guides the migratory border cells to the oocyte (for review see [60], Table 2). 

EGFR signals via the canonical Ras/MAPK to activate the transcription of target genes in a context-dependent manner via the Pointed ETS transcription factor [61]. Its expression during development is broad and therefore is not considered to be the critical aspect regulating its activity [62]. Instead, this responsibility mostly falls to the regulation of its ligands. The *Drosophila* genome encodes four ligands for EGFR; three that are produced as transmembrane precursors, Spitz, Keren, and Gurken, and one that is constitutively secreted called Vein [63]. Spitz has been most extensively characterized since it is involved in most of the EGFR-mediated processes, particularly during embryogenesis. 

Surprisingly, like EGFR, Spitz expression is also broad. This is because its activity (and that of Keren and Gurken) further requires proteolytic processing for membrane release and secreted ligand activity [118,119]. At the center of this mechanism is Rhomboid, the founding member of a serine-protease superfamily that acts via intramembrane cleavage [55]. Mutants of *rhomboid* (*rho*) phenocopy loss of *spitz*, and the highly dynamic expression pattern of *rho* closely mirrors that of EGFR-induced MAPK activation, suggesting that *rho* expression is a critical localizing determinant of EGFR signaling [120]. 

In several contexts, *rho* is also a transcriptional target of EGFR signaling. Induction of *rho* in signal receiving cells therefore converts them into a signal source. Since EGFR ligands predominantly act at close range (one to two cells away), this mechanism permits expansion of the signal and can generate complex tissue patterning such as during compound eye development [121]. During ovarian follicle cell patterning this mechanism is used to relay EGFR activity across the epithelia [120,122].

Studies of *Drosophila* EGFR activity have also described the use of negative feedback circuits to produce binary switch-like outputs. The most well studied of these involves the transcriptional target *argos,* which encodes a secreted EGFR mimetic that acts to sequester and inhibit secreted Spitz [48,123,124]. Consistent with its important role in regulating EGFR signaling, loss of *argos* results in phenotypes resembling *Egfr* gain-of-function mutants [125]. Argos is considered to be a long-range Spitz inhibitor, the consequence of which results in a steep concentration gradient of active Spitz from its point of release, and thereby limiting the spread of the ligand. While many other factors and mechanisms have been described in the control of EGFR signaling in *Drosophila*, some operating only in particular developmental contexts [126], the EGFR system relies predominantly on localized ligand production and regulatory feedback mechanisms to achieve precise and robust developmental outcomes.

## 4. Insulin-Like Receptor

Like its mammalian counterparts, IR and IGF1R, the *Drosophila* insulin-like receptor (InR) is a major player in the control of cell, organ, and body growth [5]. InR is ubiquitously expressed with notable enrichment in neuronal tissue and ovaries [94], and is activated throughout the lifecycle in response to nutrition [54,127]. Loss of InR results in embryonic lethality with severe defects in nervous system development (e.g., neuroblast loss, [128]) and failures in germband retraction and dorsal closure [73]. More well-known, however, are the striking growth phenotypes observed in viable mutants with reduced InR activity. These flies are approximately half the size of controls as a result of cell autonomous reductions in both cell size and number yet maintain normal body proportions [129]. 

Consistent with their role as the predominant downstream transducers (and regulators) of the InR signal, reductions in Chico (homolog of the insulin receptor substrates, IRS1-4), PI3K, PTEN, Akt, TOR, and FOXO function all profoundly affect cell size and number [129,130]. The viability of *chico* nulls, however, suggests that other InR signaling substrates may also exist [131]. This is also observed in mice, where multiple IRS proteins are required for mediating insulin activity [132]. 

The *Drosophila* genome encodes eight insulin-like peptides (DILP1–8) that share structural similarity to preproinsulin (DILP1–5, [129]), IGF1 (DILP6, [133]), and the relaxin family of ILPs that instead bind GPCRs (DILP7, 8, [134,135]. The DILPs have distinct spatial and temporal expression patterns throughout development and in the adult, and consistent with their function as InR ligands, their overexpression causes increased body size [136]. Of particular importance to the control of systemic growth are DILP2, 3, and 5, which are expressed in a set of neurosecretory cells (insulin producing cells, IPCs) in the larval brain and released directly into circulation. Genetic deletion of DILP2, 3, and 5 or ablation of these neurons causes growth phenotypes very similar to *chico* nulls, suggesting that these are responsible for a large proportion InR-mediated growth [137,138]. 

The IPCs, like pancreatic beta-cells in mammals, are under tight control. IPCs sense an expanding list of different neurotransmitters and peptides from other neurons, as well as factors from the gut and other tissues that all converge on DILP regulation (for review see [135]). These inputs and others, including glucose-sensing mechanisms (direct and indirect) influence the transcription of each *Ilp* independently, as well as their translation and secretion into the lymph. For example, adipokinetic hormone (glucagon-like) signaling in the IPCs has been shown to trigger selective release of DILP3 [139]. Intriguingly, under conditions of starvation, a further layer of DILP regulation is achieved post-secretion where the circulating insulin-like growth factor binding protein (Imp-L2) binds DILP2 and dampens its insulin signaling activity [140]. Thus, systemic InR activity is controlled by the production and release of the DILPs and, to a lesser extent, their extracellular sequestration (i.e., compared to Argos in EGFR signaling).

## 5. The PDGF/VEGF Receptor

*Pvr* encodes the only known *Drosophila* ortholog of the platelet derived growth factor (PDGF) and vascular endothelial growth factor (VEGF) receptor families. Akin to its mammalian relatives, Pvr comprises an ectodomain with seven immunoglobulin-like repeats and a split intracellular TKD [141]. Many functional similarities exist between mammalian VEGFRs and PDGFRs and *Drosophila* Pvr. For instance, PDGFR and VEGFR families are critical for hematopoiesis and blood vessel formation, respectively in mammals (for review see [142,143], while in flies, Pvr is required for a host of processes, including the migration of embryonic blood cells called hemocytes [144,145,146], the survival and proliferation of hemocytes and glial cells [7,147], and morphogenesis of vascular-like tubular structures such as the Malpighian tubules (kidney-like organs) and the salivary gland [148,149]. These striking similarities suggest that the ancestral function of these RTK families may have been in hematopoiesis rather than blood vessel formation [144]. Pvr is also critical for other events such as the proliferation of adult midgut stem cells [150] and the collective migration of ovarian border cells during oogenesis [74].

There are three known ligands for Pvr, Pvf1–3, all of which share a mammalian VEGF-like domain architecture defined by a centrally located PDGF/VEGF domain comprising a cysteine-knot motif [144]. In order to bind their cognate receptors, mammalian VEGFs require extensive proteolytic processing that can yield a complex population of dimerized precursors and mature forms [151]. It is not yet known whether these aspects of control are shared with *Drosophila* Pvfs.

Unlike *Egfr* and *InR*, *Pvr* is not broadly expressed. In the embryo, *Pvr* expression is restricted to midline glia of the central nervous system and mature hemocytes [75]. Hemocytes are macrophage-like phagocytes that resemble cells of the vertebrate myeloid lineage and are necessary for clearance of cellular debris (e.g., from programmed cell death) and pathogens, wound healing, and the deposition of basement membrane [7,145]. Here, Pvr plays dual roles; it is necessary for both hemocyte survival and dispersal throughout the embryo. Hemocytes first differentiate in the head mesoderm before moving posteriorly via both open and invasive migratory routes [152,153]. Expression of *Pvf2* and *Pvf3* correlate with the paths taken by hemocytes, and their mutation (removal of both genes) causes greatly reduced hemocyte numbers and defective dispersal patterns [7,144]. Initially it was thought that, in addition to their roles as trophic factors, Pvf2 and Pvf3 were chemoattractants [146]. However, more recent data suggest that these ligands are not required for guidance to the barrier, but rather for driving invasion once there [152]. 

**Figure 2:**
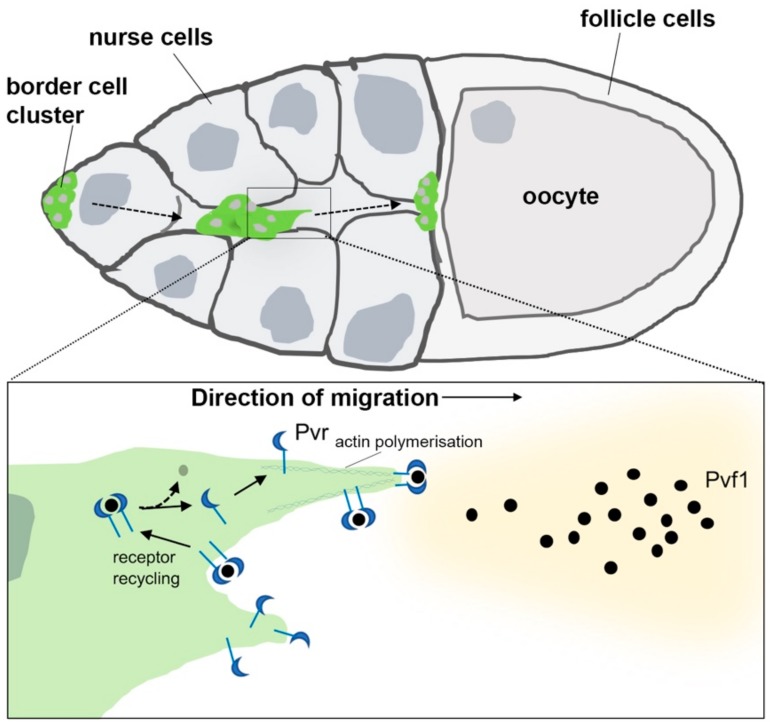
Pvr-Pvf1 mediated collective migration of border cells in the *Drosophila* ovary. Pvf1 emanating from the posteriorly located oocyte stimulates the collective migration of the border cell cluster toward its anterior boundary during oogenesis. Activation of Pvr by Pvf1 (black dots) at the leading-edge drives actin polymerization within invasive foci (inset). This is maintained by local receptor recycling (solid arrows) following endocytosis, complex disassembly (dotted arrow), then trafficking back to the cell surface.

Another informative migratory event involving Pvr permits a small cluster of ovarian follicle cells, the border cells, to migrate to the anterior end of the oocyte during oogenesis [74]. This process depends upon the partially redundant activities of EGFR and Pvr signaling in the border cells that are guided to the oocyte by their respective ligands, Gurken and Pvf1 [74]. Despite these cells showing evidence of MAPK activation during migration, the instructive cue is signaled via the Rac GTPase and its activator Myoblast-city to organize actin and polarize the recipient cell. In an elegant study, Jekely et al. [52] manipulated EGFR and Pvr levels in border cells and demonstrated that the location of the receptor at the leading edge within the cells is the most critical parameter for their guidance. This appears to be facilitated by the endocytosis and recycling of active Pvr/EGFR receptors, since inhibiting receptor endocytosis induced loss of localized signaling and severe migration defects (Figure 2). 

## 6. Torso in Embryonic Patterning and the Initiation of Metamorphosis

Torso (Tor) was identified in the seminal mutagenesis screens of the 1980s as one of a handful of maternal factors critical for specifying cell fate at the embryonic termini [64,65]. Loss of function mutations in these genes all share a common phenotype: A defective head skeleton and absence of segments posterior to abdominal segment A7 [154]. Positional cloning and sequencing of *tor* and other terminal class genes revealed that terminal patterning is the result of RTK signaling. 

**Figure 3:**
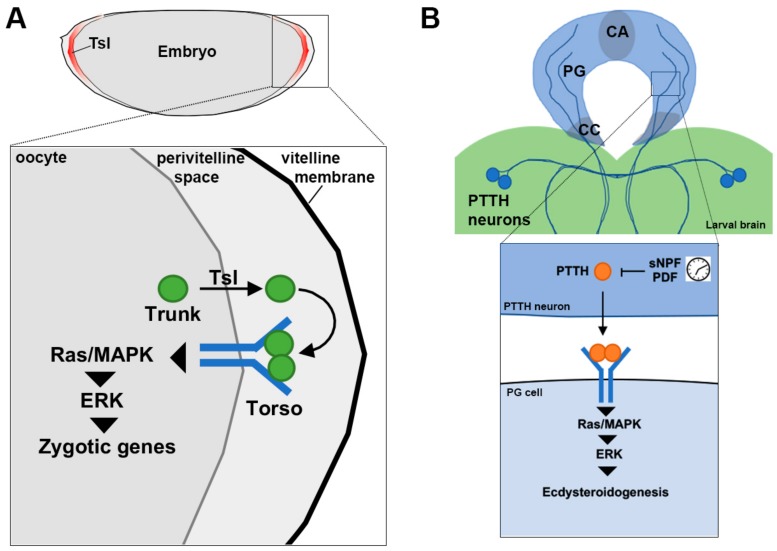
Torso signaling in embryonic patterning and the timing of developmental transitions. (**A**) Activation of Torso by its ligand Trunk at the termini of the early embryo triggers the de-repression of zygotic target genes and the specification of terminal cell fate. Torso signaling location is determined by Torso-like (Tsl), which is present only at the termini and is thought to permit the release of Trunk into the perivitelline space. (**B**) During larval development, Torso is activated by a second ligand, called PTTH, to trigger development transitions, including the initiation of metamorphosis. Torso is expressed in the major endocrine organ, the prothoracic gland (PG), which is directly innervated by two pairs of PTTH producing neurons from the larval brain. PTTH production and/or release is gated by clock neurons. Mitogen-activated protein kinase, MAPK; extracellular signal regulated kinase, ERK; corpus allatum, CA; prothoracic gland, PG; corpora cardiaca, CC; prothoracicotropic hormone, PTTH; short neuropeptide F, sNPF; pigment dispersing factor, PDF.

Like other maternal gene products, the mRNA that encodes Tor is deposited into the developing oocyte during oogenesis and translated upon fertilization where it is then thought to be present ubiquitously on the early embryo plasma membrane [65]. The ligand for Tor is encoded by *trunk* (*trk*), a member of the cysteine-knot family of growth factors and cytokines, and whose expression mirrors that of Tor [155]. Despite both Tor and Trk being present throughout the embryo, Tor is only activated at the termini. Localized activation of Tor is achieved by the function of a third protein called Torso-like (Tsl, [156], Figure 3A).

Tsl is localized to the inside of the innermost layer of the eggshell coinciding with the domain of Tor activation as measured by activation of ERK and its zygotic transcriptional targets *tailless* and *huckebein* [157,158]. Loss of *tsl* causes terminal patterning defects identical to loss of *trk* and *tor*, and when *tsl* is ectopically expressed beyond the termini, ectopic Tor activity is observed [159]. Early work suggested that once produced at the termini, limiting amounts of the Tor ligand were seized by Tor, and in the absence of Tor, the ligand could diffuse freely in the extracellular space [64,160]. These studies established the Tor/terminal patterning system as a tractable model for revealing spatial control mechanisms that continues to provide insights, despite that both RTK-mediated terminal patterning and Tor itself are not highly conserved [46,161,162].

Based on protein sequence Tsl is not predicted to have a signaling function. Instead, Tsl is a member of the membrane attack complex/perforin (MACPF) protein superfamily known for their roles as pore-forming effectors in vertebrate immunity [163,164]. We and others have been interested to understand how Tsl permits activation of Trk/Tor signaling [165]. One hypothesis is that Tsl has mediates proteolytic cleavage of Trk to allow it to bind Tor [166]. However, proteolytic processing of Trk appears to be independent of Tsl and cell culture experiments suggest that this takes place intracellularly (mediated by prohormone convertases, Furins 1 and 2 [167]). An alternative hypothesis that has found support is that Tsl stimulates localized secretion of Trk at the termini, possibly via the formation of transient membrane pores [167,168]. 

If Trk matures into the Tor ligand prior to secretion, it would presumably require physical separation from Tor during its trafficking to the membrane to avoid Tsl-independent, and therefore unrestricted, activation. Alternatively, Trk may require other factors (i.e., chaperones) or post-translational modifications before being capable of binding Tor. Whether such events are linked to the function of Tsl remain to be known. Interestingly, there are MACPF proteins in vertebrates that are also involved in developmental events (for review see [169]). Understanding how these and Tsl work may reveal common new mechanisms for cell signaling control. 

Tor has a second function during late larval development in the major endocrine gland, the prothoracic gland, that when activated acts as an instructional cue for the initiation of metamorphosis [98]. Tor signaling via Ras/MAPK leads to the synthesis and release of 20-hydroxyecdysone, a critical insect hormone for moulting. In this role, the ligand for Tor is encoded by prothoracicotropic hormone (PTTH), a cysteine-knot growth factor closely related to Trk [161]. Unlike the terminal system however, PTTH is not produced in the same cell type as Tor. PTTH is produced in a small number of neurons in the larval brain that innervate the prothoracic gland cells directly [170] (Figure 3B). Its activity appears to be mediated by its transcription, which is governed by inputs from other neurons, including those that control circadian rhythm [170,171]. The reason why Tor signaling via Trk requires Tsl, but PTTH does not remains to be determined. Recent structural insights into the interaction between Tor and PTTH have suggested that different signaling outcomes could be achieved by tuning receptor levels [172]. It will be interesting to learn whether Trk interacts with Tor in a similar manner to PTTH given its action in a developmental context with seemingly very distinct temporal requirements. 

## 7. Perspectives

Studies of RTKs and their pathways in model organisms such as *Drosophila* have and continue to yield important insights into the components and mechanisms that control them. Importantly, this work has provided an appreciation for how decisions of cell fate are determined in the context of development and how disease arises when such processes fail. However, there is still much to learn. For example, we know very little regarding how the dynamics of RTK activation (and deactivation) contributes to cell-fate decisions and the role that their ligands play in this. How important is it that RTK signaling occurs at a particular time or place, or at a level or duration sufficient to ensure a cell-fate decision is made? Answers to these questions requires advances in quantitative in vivo approaches that permit precise measurements of signaling activity and the ability to modulate RTK signaling in native developmental contexts.

Still, the most widely employed approach to measuring RTK signaling output in vivo uses sample fixation and antibodies raised against phosphorylated (active) signaling substrates, such as ERK and Akt (e.g., [173,174,175]). Unfortunately, however, the development of live-imaging approaches to measure RTK signaling has been more challenging. The most successful strategies employed to date use Förster resonance energy transfer (FRET)-based sensors (e.g., see [176,177]), which have been highly informative and sensitive in cell-culture systems. While their translation to transgenic models for in vivo use have not been as successful, several recent examples using FRET, as well as other approaches, including measuring nuclear-to-cytoplasmic ratio changes in fluorescent sensors, have shown promise [178,179]. 

With respect to RTK manipulation, the last few years have seen the emergence of optogenetics for controlling RTK activity and that of their signaling pathways [180,181,182]. This technique uses light-sensitive protein domains to induce subcellular localization changes or dimerization between two proteins (for review see [183]). Recently, several groups have successfully employed optogenetic approaches to modulate RTKs and their pathways in *Drosophila* (e.g., Tor, Ret pathways [162,182,184]). When coupled with the power of classical genetics available in model organisms, this technique offers the ability to begin dissecting the influence of critical signaling parameters, such as RTK activation amplitude and duration, as well as spatial and temporal activity on specific cell fates. Such information will entitle us to a greater understanding of the functional and mechanistic differences between RTK families and may help to explain why developmental processes have evolved to use particular RTKs and not others.

## Figures and Tables

**Table 1:** *Drosophila* RTKs and their respective mammalian subfamilies.

Mammalian RTK Family Symbol	Mammalian RTK Family Members	*Drosophila* RTK Homolog
ALK	ALK, LTK	Anaplastic lymphoma kinase (Alk)
FGFR	FGFR(1–4)	Breathless (Btl), Heartless (Htl)
DDR1 and DDR2	DDR1, DDR2	Discoidin domain receptor (Ddr)
RYK	RYK	Doughnut on 2 (Dnt), Derailed (Drl)^†^, Derailed 2 (Drl-2)
EGFR	EGFR, ERBB(2–4)	Epidermal growth factor receptor (EGFR)
EPH	EphA(1–8), EphA10	Erythropoietin-producing human hepatocellular carcinoma cell line (Eph)
INSR/IGF1R	INSR, IGF1R, IGF2R	Insulin-like receptor (Inr)
MuSK	MuSK	Neurotrophic receptor kinase (Nrk)
TRK	TrkA, TrkB, TrkC	Offtrack (Otk)^†^
PDGFR	PDGFRα, PDGFR β, Kit, CSF-1R	PDGF- and VEGF-receptor related (Pvr)
VEGFR	VEGFR(1–3)	PDGF- and VEGF-receptor related (Pvr)
RET	RET	Ret oncogene (Ret)
ROR1 and ROR2	ROR1, ROR2	RTK-like orphan receptors (Ror)
TIE	TIE1, TIE 2	Tie-like receptor (Tie)
		Sevenless (Sev)
		Torso (Tor)
		Stitcher (Cad96Ca)

^†^ Denotes an RTK with a catalytically inactive TK domain.

**Table 2:** *Drosophila* receptor tyrosine kinases and their functions during development.

Life Stage	Receptor (Gene Symbol)	Ligand (Gene Symbol)	Function	References
Embryo	*tor*	*trk*	Terminal patterning (maternal)	[64,65]
	*Egfr*	*spi, vn*	Embryonic patterning, cell recruitment, specification, proliferation, cell attachment	[60,66,67,68]
	*htl*	*pyr, ths*	Visceral mesoderm specification, migration during gastrulation (cardiogenesis)	[69,70]
	*btl*	*bnl*	Tracheal cell migration, central nervous system patterning	[71]
	*sev*	*boss*	Male gonad stem cell niche restriction	[72]
	*InR*	*Ilp2, 4, 7*	Epidermal and neural cell growth	[73]
	*Pvr*	*Pvf2, 3*	Hemocyte migration	[74,75,76]
	*dnt, drl, drl-2*	*wnt5*	Axon targeting, salivary gland cell migration	[77,78,79]
	*Alk*	*jeb*	Muscle cell specification, neuronal differentiation	[80,81]
	*Ror*	−	Neural cell growth	[82]
	*Nrk*	−	Neural cell growth^†^	[83]
	*otk*	*wnt4*	Neuroblast migration	[84]
	*Cad96Ca*	*collagen*	Wound healing, axon patterning^†^	[56]
	*Tie*	*Pvf1* ^†^	Border cell migration	[85]
Larva	*sev*	*boss*	Photoreceptor specification	[86]
	*Egfr*	*spi, vn*	Imaginal and wing disc determination, proliferation of eye disc cells	[51,87,88]
	*Pvr*	*Pvf2*	Hemocyte proliferation	[89,90]
	*htl*	*pyr, ths*	Imaginal disc determination	[91,92]
	*btl*	*bnl*	Retinal patterning and glial migration	[93]
	*InR*	*Ilps 1–6*	Neural cell and imaginal disc growth	[94]
	*Alk*	*jeb*	Spares organ growth during starvation	[95]
	*otk*	-	Photoreceptor axon guidance	[96]
	*Eph*	*Eph*	Photoreceptor axon pathfinding	[97]
	*tor*	*Ptth*	Initiation of metamorphosis	[98]
Pupa	*Egfr*	*spi, vn*	Neuron differentiation, leg patterning, specification of bract cell fate	[99,100,101]
	*Pvr*	*Pvf1, 3*	Hemocyte proliferation and differentiation, maintenance of cell adhesion	[102,103]
	*htl*	*pyr, ths*	Heart muscle remodeling, leg and thoracic muscle cell differentiation	[104,105]
	*btl*	*bnl*	Imaginal tracheoblast remodeling, eye disc remodeling, male genital disc migration	[93,106,107]
	*Alk*	*jeb*	Photoreceptor axon migration	[108]
	*Nrk*	−	Nervous system restructuring	[83]
	*Tie*	-	Retinal cell differentiation†	[109]
Adult	*Egfr*	*grk, spi*	Midgut maintenance, spermatogenesis, oogenesis, germline stem cell attenuation (testes), border cell migration (ovary)	[110,111,112,113]
	*Pvr*	*Pvf1, 2*	Midgut maintenance, border cell migration (ovary)	[74,114]
	*InR*	*Ilp2, 3, 5*	Brain, thorax, abdomen, and gut cell maintenance; body growth	[5,115]
	*drl*	*wnt5*	Axon targeting	[116]
	*Btl*	*bnl*	Axon retraction (visual system)	[117]

† Denotes a predicted function or ligand that has not been experimentally confirmed. – Denotes ligand is unknown.

## References

[1] Schlessinger J. (2014). Receptor tyrosine kinases: Legacy of the first two decades. Cold Spring Harb. Perspect. Biol..

[2] Lemmon M.A., Schlessinger J. (2010). Cell Signaling by Receptor Tyrosine Kinases. Cell.

[3] Perrimon N., Pitsouli C., Shilo B.Z. (2012). Signaling Mechanisms Controlling Cell Fate and Embryonic Patterning. CSH Perspect. Biol..

[4] Park I., Lee H.S. (2015). EphB/ephrinB Signaling in Cell Adhesion and Migration. Mol. Cells.

[5] Chen C., Jack J., Garofalo R.S. (1996). The *Drosophila* insulin receptor is required for normal growth. Endocrinology.

[6] Matsuoka S., Hiromi Y., Asaoka M. (2013). Egfr signaling controls the size of the stem cell precursor pool in the *Drosophila* ovary. Mech. Dev..

[7] Bruckner K., Kockel L., Duchek P., Luque C.M., Rorth P., Perrimon N. (2004). The PDGF/VEGF receptor controls blood cell survival in *Drosophila*. Dev. Cell.

[8] Hu K., Olsen B.R. (2016). The roles of vascular endothelial growth factor in bone repair and regeneration. Bone.

[9] Du Z., Lovly C.M. (2018). Mechanisms of receptor tyrosine kinase activation in cancer. Mol. Cancer.

[10] Ahluwalia M.S., Becker K., Levy B.P. (2018). Epidermal Growth Factor Receptor Tyrosine Kinase Inhibitors for Central Nervous System Metastases from Non-Small Cell Lung Cancer. Oncologist.

[11] Shen G.S., Zheng F.C., Ren D.F., Du F., Dong Q.X., Wang Z.Y., Zhao F.X., Ahmad R., Zhao J.D. (2018). Anlotinib: A novel multi-targeting tyrosine kinase inhibitor in clinical development. J. Hematol. Oncol..

[12] Odawara M., Kadowaki T., Yamamoto R., Shibasaki Y., Tobe K., Accili D., Bevins C., Mikami Y., Matsuura N., Akanuma Y. (1989). Human Diabetes Associated with a Mutation in the Tyrosine Kinase Domain of the Insulin-Receptor. Science.

[13] Almendro V., Garcia-Recio S., Gascon P. (2010). Tyrosine kinase receptor transactivation associated to G protein-coupled receptors. Curr. Drug Targets.

[14] Mustonen T., Alitalo K. (1995). Endothelial Receptor Tyrosine Kinases Involved in Angiogenesis. J. Cell Biol..

[15] McDonell L.M., Kernohan K.D., Boycott K.M., Sawyer S.L. (2015). Receptor tyrosine kinase mutations in developmental syndromes and cancer: Two sides of the same coin. Hum. Mol. Genet..

[16] Choura M., Rebai A. (2011). Receptor tyrosine kinases: From biology to pathology. J. Recept. Signal Transduct..

[17] Pawson T. (2002). Regulation and targets of receptor tyrosine kinases. Eur. J. Cancer.

[18] Kroiher M., Miller M.A., Steele R.E. (2001). Deceiving appearances: Signaling by “dead” and “fractured” receptor protein tyrosine kinases. Bioessays.

[19] Shi X., Hapiak V., Zheng J., Muller-Greven J., Bowman D., Lingerak R., Buck M., Wang B.C., Smith A.W. (2017). A role of the SAM domain in EphA2 receptor activation. Sci. Rep..

[20] Molina L., Marino-Buslje C., Quinn D.R., Siddle K. (2000). Structural domains of the insulin receptor and IGF receptor required for dimerisation and ligand binding. FEBS Lett..

[21] Hanks S.K., Quinn A.M., Hunter T. (1988). The Protein-Kinase Family—Conserved Features and Deduced Phylogeny of the Catalytic Domains. Science.

[22] Manning G., Plowman G.D., Hunter T., Sudarsanam S. (2002). Evolution of protein kinase signaling from yeast to man. Trends Biochem. Sci..

[23] Bae J.H., Schlessinger J. (2010). Asymmetric tyrosine kinase arrangements in activation or autophosphorylation of receptor tyrosine kinases. Mol. Cells.

[24] Shen J., Maruyama I.N. (2011). Nerve growth factor receptor TrkA exists as a preformed, yet inactive, dimer in living cells. FEBS Lett..

[25] Wehrman T., He X.L., Raab B., Dukipatti A., Blau H., Garcia K.C. (2007). Structural and mechanistic insights into nerve growth factor interactions with the TrkA and p75 receptors. Neuron.

[26] Burgess A.W., Cho H.S., Eigenbrot C., Ferguson K.M., Garrett T.P.J., Leahy D.J., Lemmon M.A., Sliwkowski M.X., Ward C.W., Yokoyama S. (2003). An open-and-shut case? Recent insights into the activation of EGF/ErbB receptors. Mol. Cell.

[27] Yayon A., Klagsbrun M., Esko J.D., Leder P., Ornitz D.M. (1991). Cell-Surface, Heparin-Like Molecules Are Required for Binding of Basic Fibroblast Growth-Factor to Its High-Affinity Receptor. Cell.

[28] Yuzawa S., Opatowsky Y., Zhang Z.T., Mandiyan V., Lax I., Schlessinger J. (2007). Structural basis for activation of the receptor tyrosine kinase KIT by stem cell factor. Cell.

[29] Pawson T., Gish G.D., Nash P. (2001). SH2 domains, interaction modules and cellular wiring. Trends Cell Biol..

[30] Hubbard S.R. (2004). Juxtamembrane autoinhibition in receptor tyrosine kinases. Nat. Rev. Mol. Cell Biol..

[31] Sun Y., Liu W.Z., Liu T., Feng X., Yang N., Zhou H.F. (2015). Signaling pathway of MAPK/ERK in cell proliferation, differentiation, migration, senescence and apoptosis. J. Recept. Signal Transduct..

[32] Krishna M., Narang H. (2008). The complexity of mitogen-activated protein kinases (MAPKs) made simple. Cell. Mol. Life Sci..

[33] Manning B.D., Cantley L.C. (2007). AKT/PKB signaling: Navigating downstream. Cell.

[34] Yuan T.L., Wulf G., Burga L., Cantley L.C. (2011). Cell-to-Cell Variability in PI3K Protein Level Regulates PI3K-AKT Pathway Activity in Cell Populations. Curr. Biol..

[35] Bunney T.D., Katan M. (2011). PLC regulation: Emerging pictures for molecular mechanisms. Trends Biochem. Sci..

[36] Shuai K. (2000). Modulation of STAT signaling by STAT-interacting proteins. Oncogene.

[37] Wittes J., Schupbach T. (2019). A Gene Expression Screen in *Drosophila* melanogaster Identifies Novel JAK/STAT and EGFR Targets During Oogenesis. G3-Genes Genomes Genet..

[38] Xu N., Wang S.Q., Tan D., Gao Y.W., Lin G.N., Xi R.W. (2011). EGFR, Wingless and JAK/STAT signaling cooperatively maintain *Drosophila* intestinal stem cells. Dev. Biol..

[39] Simon A.R., Takahashi S., Severgnini M., Fanburg B.L., Cochran B.H. (2002). Role of the JAK-STAT pathway in PDGF-stimulated proliferation of human airway smooth muscle cells. Am. J. Physiol. Lung Cell..

[40] Li W.X., Agaisse H., Mathey-Prevot B., Perrimon N. (2002). Differential requirement for STAT by gain-of-function and wild-type receptor tyrosine kinase Torso in *Drosophila*. Development.

[41] Casaletto J.B., McClatchey A.I. (2012). Spatial regulation of receptor tyrosine kinases in development and cancer. Nat. Rev. Cancer.

[42] Agarwal G., Smith A.W., Jones B. (2019). Discoidin domain receptors: Micro insights into macro assemblies. BBA-Mol. Cell Res..

[43] Delos Santos R.C., Garay C., Antonescu C.N. (2015). Charming neighborhoods on the cell surface: Plasma membrane microdomains regulate receptor tyrosine kinase signaling. Cell. Signal..

[44] Volinsky N., Kholodenko B.N. (2013). Complexity of Receptor Tyrosine Kinase Signal Processing. CSH Perspect. Biol..

[45] Simons M., Gordon E., Claesson-Welsh L. (2016). Mechanisms and regulation of endothelial VEGF receptor signalling. Nat. Rev. Mol. Cell Biol..

[46] Zinkle A., Mohammadi M. (2018). A threshold model for receptor tyrosine kinase signaling specificity and cell fate determination. F1000Research.

[47] Nakayama M., Nakayama A., van Lessen M., Yamamoto H., Hoffmann S., Drexler H.C.A., Itoh N., Hirose T., Breier G., Vestweber D. (2013). Spatial regulation of VEGF receptor endocytosis in angiogenesis. Nat. Cell Biol..

[48] Klein D.E., Nappi V.M., Reeves G.T., Shvartsman S.Y., Lemmon M.A. (2004). Argos inhibits epidermal growth factor receptor signalling by ligand sequestration. Nature.

[49] Matkar S., An C.Y., Hua X.X. (2017). Kinase inhibitors of HER2/AKT pathway induce ERK phosphorylation via a FOXO-dependent feedback loop. Am. J. Cancer Res..

[50] Zhang X.C., Lavoie G., Meant A., Aubert L., Cargnello M., Haman A., Hoang T., Roux P.P. (2017). Extracellular Signal-Regulated Kinases 1 and 2 Phosphorylate Gab2 To Promote a Negative-Feedback Loop That Attenuates Phosphoinositide 3-Kinase/Akt Signaling. Mol. Cell. Biol..

[51] Freeman M. (1996). Reiterative use of the EGF receptor triggers differentiation of all cell types in the *Drosophila* eye. Cell.

[52] Jekely G., Sung H.H., Luque C.M., Rorth P. (2005). Regulators of endocytosis maintain localized receptor tyrosine kinase signaling in guided migration. Dev. Cell.

[53] Lim B., Dsilva C.J., Levario T.J., Lu H., Schupbach T., Kevrekidis I.G., Shvartsman S.Y. (2015). Dynamics of Inductive ERK Signaling in the *Drosophila* Embryo. Curr. Biol..

[54] Oldham S., Stocker H., Laffargue M., Wittwer F., Wymann M., Hafen E. (2002). The *Drosophila* insulin/IGF receptor controls growth and size by modulating PtdInsP(3) levels. Development.

[55] Urban S., Lee J.R., Freeman M. (2001). *Drosophila* Rhomboid-1 defines a family of putative intramembrane serine proteases. Cell.

[56] Wang S.Q., Tsarouhas V., Xylourgidis N., Sabri N., Tiklova K., Nautiyal N., Gallio M., Samakovlis C. (2009). The tyrosine kinase Stitcher activates Grainy head and epidermal wound healing in *Drosophila*. Nat. Cell Biol..

[57] Cheng L., Baonza A., Grifoni D. (2018). *Drosophila* Models of Human Disease. BioMed Res. Int..

[58] Rubin G.M., Yandell M.D., Wortman J.R., Miklos G.L.G., Nelson C.R., Hariharan I.K., Fortini M.E., Li P.W., Apweiler R., Fleischmann W. (2000). Comparative genomics of the eukaryotes. Science.

[59] Kennedy S.P., Hastings J.F., Han J.Z.R., Croucher D.R. (2016). The Under-Appreciated Promiscuity of the Epidermal Growth Factor Receptor Family. Front. Cell Dev. Biol..

[60] Shilo B.Z. (2003). Signaling by the *Drosophila* epidermal growth factor receptor pathway during development. Exp. Cell Res..

[61] Vivekanand P. (2018). Lessons from *Drosophila* Pointed, an ETS family transcription factor and key nuclear effector of the RTK signaling pathway. Genesis.

[62] Zak N.B., Wides R.J., Schelter E.D., Raz E., Shilo B.Z. (1990). Localization of the Der/Flb Protein in Embryos—Implications on the Faint Little Ball Lethal Phenotype. Development.

[63] Shilo B.Z. (2005). Regulating the dynamics of EGF receptor signaling in space and time. Development.

[64] Casanova J., Struhl G. (1989). Localized Surface-Activity of Torso, a Receptor Tyrosine Kinase, Specifies Terminal Body Pattern in *Drosophila*. Genes Dev..

[65] Sprenger F., Stevens L.M., Nussleinvolhard C. (1989). The *Drosophila* Gene Torso Encodes a Putative Receptor Tyrosine Kinase. Nature.

[66] Buff E., Carmena A., Gisselbrecht S., Jimenez F., Michelson A.M. (1998). Signalling by the *Drosophila* epidermal growth factor receptor is required for the specification and diversification of embryonic muscle progenitors. Development.

[67] Duchek P., Rorth P. (2001). Guidance of cell migration by EGF receptor signaling during *Drosophila* oogenesis. Science.

[68] Skeath J.B. (1998). The *Drosophila* EGF receptor controls the formation and specification of neuroblasts along the dorsal-ventral axis of the *Drosophila* embryo. Development.

[69] Gisselbrecht S., Skeath J.B., Doe C.Q., Michelson A.M. (1996). Heartless encodes a fibroblast growth factor receptor (DFR1/DFGF-R2) involved in the directional migration of early mesodermal cells in the *Drosophila* embryo. Genes Dev..

[70] Beiman M., Shilo B.Z., Volk T. (1996). Heartless, a *Drosophila* FGF receptor homolog, is essential for cell migration and establishment of several mesodermal lineages. Genes Dev..

[71] Klambt C., Glazer L., Shilo B.Z. (1992). Breathless, a *Drosophila* Fgf Receptor Homolog, Is Essential for Migration of Tracheal and Specific Midline Glial-Cells. Genes Dev..

[72] Kitadate Y., Shigenobu S., Arita K., Kobayashi S. (2006). Boss/Sev signaling from germline to soma specify the germline stem cell niche in *Drosophila* male gonads. Zool. Sci..

[73] Fernandez R., Tabarini D., Azpiazu N., Frasch M., Schlessinger J. (1995). The *Drosophila* Insulin-Receptor Homolog—A Gene Essential for Embryonic-Development Encodes 2 Receptor Isoforms with Different Signaling Potential. EMBO J..

[74] Duchek P., Somogyi K., Jekely G., Beccari S., Rorth P. (2001). Guidance of cell migration by the *Drosophila* PDGF/VEGF receptor. Cell.

[75] Heino T.I., Karpanen T., Wahlstrom G., Pulkkinen M., Eriksson U., Alitalo K., Roos C. (2001). The *Drosophila* VEGF receptor homolog is expressed in hemocytes. Mech. Dev..

[76] McDonald J.A., Pinheiro E.M., Montell D.J. (2003). PVF1, a PDGF/VEGF homolog, is sufficient to guide border cells and interacts genetically with Taiman. Development.

[77] Callahan C.A., Muralidhar M.G., Lundgren S.E., Scully A.L., Thomas J.B. (1995). Control of Neuronal Pathway Selection by a *Drosophila* Receptor Protein-Tyrosine Kinase Family Member. Nature.

[78] Harris K.E., Beckendorf S.K. (2007). Different Wnt signals act through the Frizzled and RYK receptors during *Drosophila* salivary gland migration. Development.

[79] Oates A.C., Bonkovsky J.L., Irvine D.V., Kelly L.E., Thomas J.B., Wilks A.F. (1998). Embryonic expression and activity of doughnut, a second RYK homolog in *Drosophila*. Mech. Dev..

[80] Englund C., Loren C.E., Grabbe C., Varshney G.K., Deleuil F., Hallberg B., Palmer R.H. (2003). Jeb signals through the Alk receptor tyrosine kinase to drive visceral muscle fusion. Nature.

[81] Gouzi J.Y., Moressis A., Walker J.A., Apostolopoulou A.A., Palmer R.H., Bernards A., Skoulakis E.M.C. (2011). The Receptor Tyrosine Kinase Alk Controls Neurofibromin Functions in *Drosophila* Growth and Learning. PLoS Genet..

[82] Paganoni S., Ferreira A. (2005). Neurite extension in central neurons: A novel role for the receptor tyrosine kinases Ror1 and Ror2. J. Cell Sci..

[83] Oishi I., Sugiyama S., Liu Z.J., Yamamura H., Nishida Y., Minami Y. (1997). A novel *Drosophila* receptor tyrosine kinase expressed specifically in the nervous system—Unique structural features and implication in developmental signaling. J. Biol. Chem..

[84] Winberg M.L., Tamagnone L., Bai J.W., Comoglio P.M., Montell D., Goodman C.S. (2001). The transmembrane protein off-track associates with plexins and functions downstream of semaphorin signaling during axon guidance. Neuron.

[85] Wang X.J., Bo J.Y., Bridges T., Dugan K.D., Pan T.C., Chodosh L.A., Montell D.J. (2006). Analysis of cell migration using whole-genome expression profiling of migratory cells in the *Drosophila* ovary. Dev. Cell.

[86] Tomlinson A., Bowtell D.D.L., Hafen E., Rubin G.M. (1987). Localization of the Sevenless Protein, a Putative Receptor for Positional Information, in the Eye Imaginal Disk of *Drosophila*. Cell.

[87] Baonza A., Casci T., Freeman M. (2001). A primary role for the epidermal growth factor receptor in ommatidial spacing in the *Drosophila* eye. Curr. Biol..

[88] Kubota K., Goto S., Eto K., Hayashi S. (2000). EGF receptor attenuates Dpp signaling and helps to distinguish the wing and leg cell fates in *Drosophila*. Development.

[89] Munier A.I., Doucet D., Perrodou E., Zachary D., Meister M., Hoffmann J.A., Janeway C.A., Lagueux M. (2002). PVF2, a PDGF/VEGF-like growth factor, induces hemocyte proliferation in *Drosophila* larvae. EMBO Rep..

[90] Zettervall C.J., Anderl I., Williams M.J., Palmer R., Kurucz E., Ando I., Hultmark D. (2004). A directed screen for genes involved in *Drosophila* blood cell activation. Proc. Natl. Acad. Sci. USA.

[91] Emori Y., Saigo K. (1993). Distinct Expression of 2 *Drosophila* Homologs of Fibroblast Growth-Factor Receptors in Imaginal Disks. FEBS Lett..

[92] Franzdottir S.R., Engelen D., Yuva-Aydemir Y., Schmidt I., Aho A., Klambt C. (2009). Switch in FGF signalling initiates glial differentiation in the *Drosophila* eye. Nature.

[93] Mukherjee T., Choi I., Banerjee U. (2012). Genetic Analysis of Fibroblast Growth Factor Signaling in the *Drosophila* Eye. G3-Genes Genomes Genet..

[94] Garofalo R.S., Rosen O.M. (1988). Tissue Localization of *Drosophila*-Melanogaster Insulin-Receptor Transcripts during Development. Mol. Cell. Biol..

[95] Cheng L.Y., Bailey A.P., Leevers S.J., Ragan T.J., Driscoll P.C., Gould A.P. (2011). Anaplastic Lymphoma Kinase Spares Organ Growth during Nutrient Restriction in *Drosophila*. Cell.

[96] Cafferty P., Yu L., Rao Y. (2004). The receptor tyrosine kinase off-track is required for layer-specific neuronal connectivity in *Drosophila*. Development.

[97] Dearborn R., He Q., Kunes S., Dai Y. (2002). Eph receptor tyrosine kinase-mediated formation of a topographic map in the *Drosophila* visual system. J. Neurosci..

[98] Rewitz K.F., Yamanaka N., Gilbert L.I., O’Connor M.B. (2009). The Insect Neuropeptide PTTH Activates Receptor Tyrosine Kinase Torso to Initiate Metamorphosis. Science.

[99] Del Alamo D., Terriente J., Diaz-Benjumea F.J. (2002). Spitz/EGFr signalling via the Ras/MAPK pathway mediates the induction of bract cells in *Drosophila* legs. Development.

[100] Galindo M.I., Bishop S.A., Greig S., Couso J.P. (2002). Leg patterning driven by proximal-distal interactions and EGFR signaling. Science.

[101] Huang Z., Shilo B.Z., Kunes S. (1998). A retinal axon fascicle uses spitz, an EGF receptor ligand, to construct a synaptic cartridge in the brain of *Drosophila*. Cell.

[102] Jung S.H., Evans C.J., Uemura C., Banerjee U. (2005). The *Drosophila* lymph gland as a developmental model of hematopoiesis. Development.

[103] Rosin D., Schejter E., Volk T., Shilo B.Z. (2004). Apical accumulation of the *Drosophila* PDGF/VEGF receptor ligands provides a mechanism for triggering localized actin polymerization. Development.

[104] Dutta D., Shaw S., Maqbool T., Pandya H., VijayRaghavan K. (2005). *Drosophila* heartless acts with Heartbroken/Dof in muscle founder differentiation. PLoS Biol..

[105] Zeitouni B., Senatore S., Severac D., Aknin C., Semeriva M., Perrin L. (2007). Signalling pathways involved in adult heart formation revealed by gene expression profiling in *Drosophila*. PLoS Genet..

[106] Ahmad S.M., Baker B.S. (2002). Sex-specific deployment of FGF signaling in *Drosophila* recruits mesodermal cells into the male genital imaginal disc. Cell.

[107] Sato M., Kornberg T.B. (2002). FGF is an essential mitogen and chemoattractant for the air sacs of the *Drosophila* tracheal system. Dev. Cell.

[108] Bazigou E., Apitz H., Johansson J., Loren C.E., Hirst E.M.A., Chen P.L., Palmer R.H., Salecker I. (2007). Anterograde jelly belly and Alk receptor tyrosine kinase signaling mediates retinal axon targeting in *Drosophila*. Cell.

[109] Michaut L., Flister S., Neeb M., White K.P., Certa U., Gehring W.J. (2003). Analysis of the eye developmental pathway in *Drosophila* using DNA microarrays. Proc. Natl. Acad. Sci. USA.

[110] Buchon N., Broderick N.A., Kuraishi T., Lemaitre B. (2010). *Drosophila* EGFR pathway coordinates stem cell proliferation and gut remodeling following infection. BMC Biol..

[111] Goode S., Wright D., Mahowald A.P. (1992). The Neurogenic Locus Brainiac Cooperates with the *Drosophila* Egf Receptor to Establish the Ovarian Follicle and to Determine Its Dorsal-Vental Polarity. Development.

[112] Kiger A.A., White-Cooper H., Fuller M.T. (2000). Somatic support cells restrict germline stem cell self-renewal and promote differentiation. Nature.

[113] Parrott B.B., Hudson A., Brady R., Schulz C. (2012). Control of Germline Stem Cell Division Frequency—A Novel, Developmentally Regulated Role for Epidermal Growth Factor Signaling. PLoS ONE.

[114] Choi N.H., Kim J.G., Yang D.J., Kim Y.S., Yoo M.A. (2008). Age-related changes in *Drosophila* midgut are associated with PVF2, a PDGF/VEGF-like growth factor. Aging Cell.

[115] Veenstra J.A., Agricola H.J., Sellami A. (2008). Regulatory peptides in fruit fly midgut. Cell Tissue Res..

[116] Dura J.M., Taillebourg E., Preat T. (1995). The *Drosophila* Learning and Memory Gene Linotte Encodes a Putative Receptor Tyrosine Kinase Homologous to the Human Ryk Gene-Product. FEBS Lett..

[117] Srahna M., Leyssen M., Choi C.M., Fradkin L.G., Noordermeer J.N., Hassan B.A. (2006). A signaling network for patterning of neuronal connectivity in the *Drosophila* brain. PLoS Biol..

[118] Adrain C., Freeman M. (2014). Regulation of receptor tyrosine kinase ligand processing. Cold Spring Harb. Perspect. Biol..

[119] Steinhauer J., Liu H.H., Miller E., Treisman J.E. (2013). Trafficking of the EGFR ligand Spitz regulates its signaling activity in polarized tissues. J. Cell Sci..

[120] Wasserman J.D., Freeman M. (1998). An autoregulatory cascade of EGF receptor signaling patterns the *Drosophila* egg. Cell.

[121] Dominguez M., Wasserman J.D., Freeman M. (1998). Multiple functions of the EGF receptor in *Drosophila* eye development. Curr. Biol..

[122] Sapir A., Schweitzer R., Shilo B.Z. (1998). Sequential activation of the EGF receptor pathway during *Drosophila* oogenesis establishes the dorsoventral axis. Development.

[123] Golembo M., Schweitzer R., Freeman M., Shilo B.Z. (1996). argos transcription is induced by the *Drosophila* EGF receptor pathway to form an inhibitory feedback loop. Development.

[124] Schweitzer R., Howes R., Smith R., Shilo B.Z., Freeman M. (1995). Inhibition of *Drosophila* Egf Receptor Activation by the Secreted Protein Argos. Nature.

[125] Freeman M., Klambt C., Goodman C.S., Rubin G.M. (1992). The Argos Gene Encodes a Diffusible Factor That Regulates Cell Fate Decisions in the *Drosophila* Eye. Cell.

[126] Avraham R., Yarden Y. (2011). Feedback regulation of EGFR signalling: Decision making by early and delayed loops. Nat. Rev. Mol. Cell Biol..

[127] Nassel D.R., Liu Y.T., Luo J.N. (2015). Insulin/IGF signaling and its regulation in *Drosophila*. Gen. Comp. Endocrinol..

[128] Tatar M., Kopelman A., Epstein D., Tu M.P., Yin C.M., Garofalo R.S. (2001). A mutant *Drosophila* insulin receptor homolog that extends life-span and impairs neuroendocrine function. Science.

[129] Brogiolo W., Stocker H., Ikeya T., Rintelen F., Fernandez R., Hafen E. (2001). An evolutionarily conserved function of the *Drosophila* insulin receptor and insulin-like peptides in growth control. Curr. Biol..

[130] Junger M.A., Rintelen F., Stocker H., Wasserman J.D., Vegh M., Radimerski T., Greenberg M.E., Hafen E. (2003). The *Drosophila* forkhead transcription factor FOXO mediates the reduction in cell number associated with reduced insulin signaling. J. Biol..

[131] Bohni R., Riesgo-Escovar J., Oldham S., Brogiolo W., Stocker H., Andruss B.F., Beckingham K., Hafen E. (1999). Autonomous control of cell and organ size by CHICO, a *Drosophila* homolog of vertebrate IRS1-4. Cell.

[132] Kido Y., Nakae J., Accili D. (2001). Clinical review 125—The insulin receptor and its cellular targets. J. Clin. Endocrinol. Metab..

[133] Slaidina M., Delanoue R., Gronke S., Partridge L., Leopold P. (2009). A *Drosophila* Insulin-like Peptide Promotes Growth during Nonfeeding States. Dev. Cell.

[134] Colombani J., Andersen D.S., Leopold P. (2012). Secreted Peptide Dilp8 Coordinates *Drosophila* Tissue Growth with Developmental Timing. Science.

[135] Nassel D.R., Vanden Broeck J. (2016). Insulin/IGF signaling in *Drosophila* and other insects: Factors that regulate production, release and post-release action of the insulin-like peptides. Cell. Mol. Life Sci..

[136] Ikeya T., Galic M., Belawat P., Nairz K., Hafen E. (2002). Nutrient-dependent expression of insulin-like peptides from neuroendocrine cells in the CNS contributes to growth regulation in *Drosophila*. Curr. Biol..

[137] Gronke S., Clarke D.F., Broughton S., Andrews T.D., Partridge L. (2010). Molecular Evolution and Functional Characterization of *Drosophila* Insulin-Like Peptides. PLoS Genet..

[138] Rulifson E.J., Kim S.K., Nusse R. (2002). Ablation of insulin-producing neurons in flies: Growth and diabetic phenotypes. Science.

[139] Kim J., Neufeld T.P. (2015). Dietary sugar promotes systemic TOR activation in *Drosophila* through AKH-dependent selective secretion of Dilp3. Nat. Commun..

[140] Honegger B., Galic M., Kohler K., Wittwer F., Brogiolo W., Hafen E., Stocker H. (2008). Imp-L2, a putative homolog of vertebrate IGF-binding protein 7, counteracts insulin signaling in *Drosophila* and is essential for starvation resistance. J. Biol..

[141] Shibuya M. (2013). VEGFR and type-V RTK activation and signaling. Cold Spring Harb. Perspect. Biol..

[142] Andrae J., Gallini R., Betsholtz C. (2008). Role of platelet-derived growth factors in physiology and medicine. Genes Dev..

[143] Shibuya M. (2013). Vascular endothelial growth factor and its receptor system: Physiological functions in angiogenesis and pathological roles in various diseases. J. Biochem..

[144] Cho N.K., Keyes L., Johnson E., Heller J., Ryner L., Karim F., Krasnow M.A. (2002). Developmental control of blood cell migration by the *Drosophila* VEGF pathway. Cell.

[145] Olofsson B., Page D.T. (2005). Condensation of the central nervous system in embryonic *Drosophila* is inhibited by blocking hemocyte migration or neural activity. Dev. Biol..

[146] Wood W., Faria C., Jacinto A. (2006). Distinct mechanisms regulate hemocyte chemotaxis during development and wound healing in *Drosophila* melanogaster. J. Cell Biol..

[147] Learte A.R., Forero M.G., Hidalgo A. (2008). Gliatrophic and gliatropic roles of PVF/PVR signaling during axon guidance. Glia.

[148] Bunt S., Hooley C., Hu N., Scahill C., Weavers H., Skaer H. (2010). Hemocyte-secreted type IV collagen enhances BMP signaling to guide renal tubule morphogenesis in *Drosophila*. Dev. Cell.

[149] Harris K.E., Schnittke N., Beckendorf S.K. (2007). Two ligands signal through the *Drosophila* PDGF/VEGF receptor to ensure proper salivary gland positioning. Mech. Dev..

[150] Bond D., Foley E. (2012). Autocrine Platelet-derived Growth Factor-Vascular Endothelial Growth Factor Receptor-related (Pvr) Pathway Activity Controls Intestinal Stem Cell Proliferation in the Adult *Drosophila* Midgut. J. Biol. Chem..

[151] Joukov V., Sorsa T., Kumar V., Jeltsch M., ClaessonWelsh L., Cao Y.H., Saksela O., Kalkkinen N., Alitalo K. (1997). Proteolytic processing regulates receptor specificity and activity of VEGF-C. EMBO J..

[152] Parsons B., Foley E. (2013). The *Drosophila* Platelet-derived Growth Factor and Vascular Endothelial Growth Factor-Receptor Related (Pvr) Protein Ligands Pvf2 and Pvf3 Control Hemocyte Viability and Invasive Migration. J. Biol. Chem..

[153] Siekhaus D., Haesemeyer M., Moffitt O., Lehmann R. (2010). RhoL controls invasion and Rap1 localization during immune cell transmigration in *Drosophila*. Nat. Cell Biol..

[154] Strecker T.R., Halsell S.R., Fisher W.W., Lipshitz H.D. (1989). Reciprocal Effects of Hyperactivity and Hypoactivity Mutations in the *Drosophila* Pattern Gene Torso. Science.

[155] Casanova J., Furriols M., McCormick C.A., Struhl G. (1995). Similarities between trunk and spatzle, putative extracellular ligands specifying body pattern in *Drosophila*. Genes Dev..

[156] Savant-Bhonsale S., Montell D.J. (1993). Torso-Like Encodes the Localized Determinant of *Drosophila* Terminal Pattern-Formation. Genes Dev..

[157] Jimenez G., Gonzalez-Reyes A., Casanova J. (2002). Cell surface proteins Nasrat and Polehole stabilize the Torso-like extracellular determinant in *Drosophila* oogenesis. Genes Dev..

[158] Stevens L.M., Beuchle D., Jurcsak J., Tong X.L., Stein D. (2003). The *Drosophila* embryonic patterning determinant torsolike is a component of the eggshell. Curr. Biol..

[159] Martin J.R., Raibaud A., Ollo R. (1994). Terminal Pattern Elements in *Drosophila* Embryo Induced by the Torso-Like Protein. Nature.

[160] Sprenger F., Nusslein Volhard C. (1992). Torso Receptor Activity Is Regulated by a Diffusible Ligand Produced at the Extracellular Terminal Regions of the *Drosophila* Egg. Cell.

[161] Duncan E.J., Benton M.A., Dearden P.K. (2013). Canonical terminal patterning is an evolutionary novelty. Dev. Biol..

[162] Goyal Y., Schupbach T., Shvartsman S.Y. (2018). A quantitative model of developmental RTK signaling. Dev. Biol..

[163] Ponting C.P. (1999). Chlamydial homologues of the MACPF (MAC/perforin) domain. Curr. Biol..

[164] Rosado C.J., Buckle A.M., Law R.H.P., Butcher R.E., Kan W.T., Bird C.H., Ung K., Browne K.A., Baran K., Bashtannyk-Puhalovich T.A. (2007). A common fold mediates vertebrate defense and bacterial attack. Science.

[165] Johnson T.K., Henstridge M.A., Warr C.G. (2017). MACPF/CDC proteins in development: Insights from *Drosophila* torso-like. Semin. Cell Dev. Biol..

[166] Casali A., Casanova J. (2001). The spatial control of Torso RTK activation: A C-terminal fragment of the Trunk protein acts as a signal for Torso receptor in the *Drosophila* embryo. Development.

[167] Johnson T.K., Henstridge M.A., Herr A., Moore K.A., Whisstock J.C., Warr C.G. (2015). Torso-like mediates extracellular accumulation of Furin-cleaved Trunk to pattern the *Drosophila* embryo termini. Nat. Commun..

[168] Mineo A., Fuentes E., Furriols M., Casanova J. (2018). Holes in the Plasma Membrane Mimic Torso-Like Perforin in Torso Tyrosine Kinase Receptor Activation in the *Drosophila* Embryo. Genetics.

[169] Kondos S.C., Hatfaludi T., Voskoboinik I., Trapani J.A., Law R.H.P., Whisstock J.C., Dunstone M.A. (2010). The structure and function of mammalian membrane-attack complex/perforin-like proteins. Tissue Antigens.

[170] McBrayer Z., Ono H., Shimell M., Parvy J.P., Beckstead R.B., Warren J.T., Thummel C.S., Dauphin-Villemant C., Gilbert L.I., O’Connor M.B. (2007). Prothoracicotropic hormone regulates developmental timing and body size in *Drosophila*. Dev. Cell.

[171] Selcho M., Millan C., Palacios-Munoz A., Ruf F., Ubillo L., Chen J., Bergmann G., Ito C., Silva V., Wegener C. (2017). Central and peripheral clocks are coupled by a neuropeptide pathway in *Drosophila*. Nat. Commun..

[172] Jenni S., Goyal Y., von Grotthuss M., Shvartsman S.Y., Klein D.E. (2015). Structural Basis of Neurohormone Perception by the Receptor Tyrosine Kinase Torso. Mol. Cell.

[173] Coppey M., Boettiger A.N., Berezhkovskii A.M., Shvartsman S.Y. (2008). Nuclear trapping shapes the terminal gradient in the *Drosophila* embryo. Curr. Biol..

[174] Helman A., Cinnamon E., Mezuman S., Hayouka Z., Von Ohlen T., Orian A., Jimenez G., Paroush Z. (2011). Phosphorylation of Groucho Mediates RTK Feedback Inhibition and Prolonged Pathway Target Gene Expression. Curr. Biol..

[175] Vinayagam A., Kulkarni M.M., Sopko R., Sun X.Y., Hu Y.H., Nand A., Villalta C., Moghimi A., Yang X.M., Mohr S.E. (2016). An Integrative Analysis of the InR/PI3K/Akt Network Identifies the Dynamic Response to Insulin Signaling. Cell Rep..

[176] Harvey C.D., Ehrhardt A.G., Cellurale C., Zhong H.N., Yasuda R., Davis R.J., Svoboda K. (2008). A genetically encoded fluorescent sensor of ERK activity. Proc. Natl. Acad. Sci. USA.

[177] Swift J.L., Godin A.G., Dore K., Freland L., Bouchard N., Nimmo C., Sergeev M., De Koninck Y., Wiseman P.W., Beaulieu J.M. (2011). Quantification of receptor tyrosine kinase transactivation through direct dimerization and surface density measurements in single cells. Proc. Natl. Acad. Sci. USA.

[178] De la Cova C., Townley R., Regot S., Greenwald I. (2017). A Real-Time Biosensor for ERK Activity Reveals Signaling Dynamics during C. elegans Cell Fate Specification. Dev. Cell.

[179] Kim J., Lee S., Jung K., Oh W.C., Kim N., Son S., Jo Y., Kwon H.B., Heo W.D. (2019). Intensiometric biosensors visualize the activity of multiple small GTPases in vivo. Nat. Commun..

[180] Leopold A.V., Chernov K.G., Shemetov A.A., Verkhusha V.V. (2019). Neurotrophin receptor tyrosine kinases regulated with near-infrared light. Nat. Commun..

[181] Goglia A.G., Wilson M.Z., DiGiorno D.B., Toettcher J.E. (2017). Optogenetic Control of Ras/Erk Signaling Using the Phy-PIF System. Methods Mol. Biol..

[182] Grusch M., Schelch K., Riedler R., Reichhart E., Differ C., Berger W., Ingles-Prieto A., Janovjak H. (2014). Spatio-temporally precise activation of engineered receptor tyrosine kinases by light. EMBO J..

[183] Krueger D., Izquierdo E., Viswanathan R., Hartmann J., Cartes C.P., De Renzis S. (2019). Principles and applications of optogenetics in developmental biology. Development.

[184] Johnson H.E., Toettcher J.E. (2018). Illuminating developmental biology with cellular optogenetics. Curr. Opin. Biotechnol..

